# Infant activity and sleep behaviors in a maternal and infant home visiting project among rural, southern, African American women

**DOI:** 10.1186/s40748-018-0078-0

**Published:** 2018-05-16

**Authors:** Jessica L. Thomson, Lisa M. Tussing-Humphreys, Melissa H. Goodman, Alicia S. Landry

**Affiliations:** 1 United States Department of Agriculture, Agricultural Research Service, 141 Experiment Station Road, Stoneville, MS 38776 USA; 2 Department of Medicine and Cancer Center, University of Illinois at Chicago, Chicago, IL USA; 3 Department of Family and Consumer Sciences, University of Central Arkansas, Conway, AR USA

**Keywords:** Infant, Non-confinement time, Sleep duration, African American, Home visiting

## Abstract

**Background:**

Physical inactivity and inadequate amounts of sleep are two potential causes for excessive weight gain in infancy. Thus, parents and caregivers of infants need to be educated about decreasing infant sedentary behavior, increasing infant unrestrained floor time, as well as age specific recommended amounts of sleep for infants. The aims of this study were to determine if maternal knowledge about infant activity and sleep changed over time and to evaluate maternal compliance rates with expert recommendations for infant sleep in a two-arm, randomized, controlled, comparative impact trial.

**Methods:**

Pregnant women at least 18 years of age, less than 19 weeks pregnant, and residing in a lower Mississippi Delta county were recruited between March 2013 and December 2014. Postnatal data was collected from 54 participants between September 2013 and May 2016. McNemar’s test of symmetry was used to determine if maternal knowledge changed over time, while generalized linear mixed models and Kaplan-Meier survival curves were used to assess compliance with expert recommendations for infant sleep.

**Results:**

The postnatal retention rate was 85%. Maternal knowledge significantly increased for correct infant sleep position (back) and beginning tummy time by one month of age. Odds of meeting sleep duration recommendations increased by 30% for every one month increase in infant age. Only 20% of the participants were compliant with the back to sleep recommendation for the first 12 months of their infant’s life; median time to noncompliance was 7.8 months.

**Conclusions:**

Although baseline knowledge concerning infant activity and sleep was high in this cohort of rural, Southern, African American mothers, compliance with recommendations was not optimal.

**Trial registration:**

The study was registered at clinicaltrials.gov (NCT01746394) on December 5, 2012.

## Background

The prevalence of excessive body weight in infants and toddlers, defined as weight for recumbent length at or above the 95th percentile on the 2000 Centers for Disease Control and Prevention sex specific growth charts, is approximately 8% in the United States (US) [1]. This is a concern because early excessive weight gain in infancy leads to increased risks of childhood obesity [2] and adult obesity, notably in African Americans [3]. One potential cause of excessive weight gain in infants is physical inactivity. Methods to increase infant physical activity include limiting the use of devices that restrict movement (e.g., car seats, bouncy seats, and swings) and providing infants with opportunities to move freely. Tummy time, defined as awake and supervised positioning on the stomach, is a form of expert recommended physical activity that allows infants to engage in unrestricted movement necessary to strengthen muscles needed for motor development [4]. Thus, it is important for parents and caregivers to recognize the need to decrease sedentary behavior in infants and increase unrestrained floor time, especially in a prone position (e.g., tummy time).

Similar to physical activity, sleep is a basic requirement for healthy development in infants. An inadequate amount of sleep in children has been associated with higher stress and anxiety, difficulty concentrating, and lower academic performance and quality of life/well-being [5]. Further, an inverse association between sleep duration in children and risk of overweight/obesity was found in a meta-analysis of prospective cohort studies [6]. Similarly, evidence from a recent review suggests a link between short sleep duration and insulin resistance in children, potentially leading to type 2 diabetes [7]. Given these adverse associations between short sleep duration and health risks in children, it is essential that parents and caregivers are educated about age specific recommendations for sleep amounts. Educating parents and caregivers about methods for improving sleep quality and increasing sleep duration (e.g., establishing a consistent bedtime routine) [8] also may be necessary to lessen the effects of poor sleep in infancy.

While recent attention has focused on activity and sleep in infants in general, less is known about these behaviors in specific US populations, such as rural, Southern, African Americans. Analysis of secondary and tertiary outcomes in a maternal, infant and early childhood home visiting project afforded such an opportunity. The Delta Healthy Sprouts Project was designed to test the comparative impact of two maternal, infant, and early childhood home visiting curricula on weight status, dietary intake, physical activity, and other health behaviors of women and their infants residing in the rural Lower Mississippi Delta region of the US [9]. Infant activity was a secondary health outcome targeted for improvement. However, preliminary analyses indicated that differences between treatment arms were not significant, likely because the importance of infant activity (e.g., “tummy time” or placing an infant on his or her stomach while awake and supervised) was discussed in both curriculums. Hence, activity and sleep outcomes of the Delta Healthy Sprouts participants’ infants, without regard to treatment arm, are described in this paper. Specifically, the objectives were to determine if maternal knowledge and beliefs concerning infant activity and sleep changed over time and to evaluate maternal compliance rates with expert recommendations for infant sleep. The secondary objectives were to explore associations among maternal knowledge and beliefs about infant activity and sleep, and infant activity and sleep behaviors.

## Methods

### Design

Delta Healthy Sprouts was a randomized two-arm parallel controlled trial. It was designed to evaluate the impact of the Parents as Teachers® (PAT, control) curriculum as compared with a nutrition and physical activity enhanced PAT curriculum (PATE, experimental) on the primary outcomes of maternal gestational weight gain and postpartum weight control and childhood obesity prevention. Participants were randomly assigned to one of the two treatment arms [PAT (*N* = 43) or PATE (*N* = 39)] using a random generator function in SAS® (version 9.4, SAS Institute Inc., Cary, NC) and equal allocation in blocks of 25. Participants were followed for 18 months, starting at approximately 4 months gestation through 12 months postnatal. The Delta State University Institutional Review Board approved the study protocol (number 12–024) and all participants provided written informed consent. The study is registered at clinicaltrials.gov (NCT01746394).

### Participants and setting

Recruitment occurred via passive (distribution of flyers and brochures) and active (study staff on site) methods at local health clinics and medical facilities serving pregnant women and at local health fairs. Women also were referred to the study by health clinic/department staff, Special Supplemental Program for Women, Infants, and Children (WIC) nutritionists, social service agencies, and enrolled study participants. Inclusion criteria included: at least 18 years of age; less than 19 weeks pregnant with first, second or third child; singleton pregnancy; and resident of Washington, Bolivar, or Humphreys County in Mississippi. Participant enrollment occurred on a rolling basis; hence baseline data were collected from 82 pregnant women between March 2013 and December 2014.

The target enrollment was 75 women in each of the two arms (control and experimental) of the project. The sample size of 150 women was based on the following assumptions: 20% attrition rate, 15% of infants in the control arm classified as obese in their first year of life, and a 12% difference between treatment arms for percentage of infants classified as obese during their first year of life. Power and sample size calculations for gestational weight gain within the Institute of Medicine recommendations and 12-month postnatal maternal weight loss also were performed [9]. Recruitment was stopped by the study’s Principal Investigator prior to reaching these numbers due to unexpected difficulties recruiting pregnant women meeting study criteria. Recruitment was extended as long as possible, but fiscal reasons eventually necessitated the closing of this period. Data collection was completed in May 2016.

**Fig. 1:**
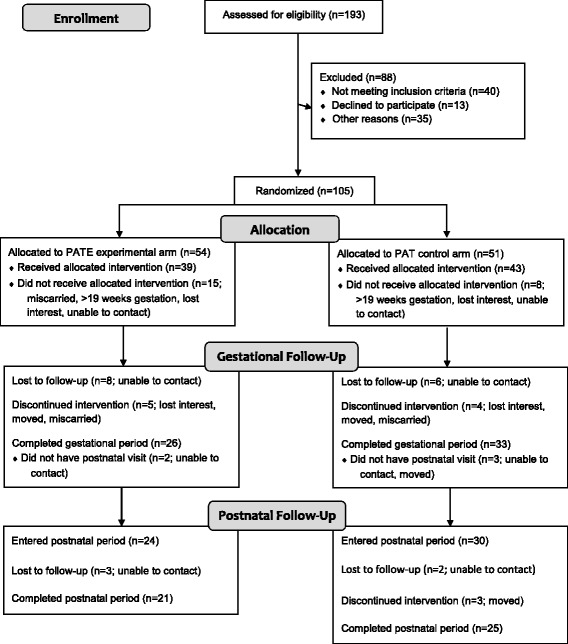
Flow diagram of recruitment, assignment, enrollment, and completion of gestational and postnatal periods for Delta Healthy Sprouts participants in the two treatment arms

Because infant activity and sleep outcomes were the primary focus of this paper, analyses were conducted only for the postnatal cohort (participants who completed the gestational period and had at least one visit in the postnatal period; *n* = 54). Five participants who completed the gestational period but dropped out of the study prior to the postnatal month (PM) 1 visit were excluded from the postnatal cohort. Fig. 1 illustrates the flow of Delta Healthy Sprouts participants through all phases of the study.

### Intervention

The PAT control arm of the intervention followed the nationally recognized, evidence-based Parents as Teachers® curriculum which included one-on-one home visits, optional monthly group meetings, developmental screenings, and a resource network for families. The Program seeks to increase parental knowledge of child development, improve parenting practices, provide early detection of developmental delays, prevent child abuse, and increase school readiness [10]. Using the PAT model, Parent Educators provided mothers with evidence-based information and activities during home visits. Materials were responsive to parental information requests and were tailored to the age of the child (or gestational age of the fetus). Specific to the analyses presented in this paper, Parent Educators discussed putting an infant to sleep on his/her back until 1 year of age, establishing a regular bedtime routine between 2 to 6 months of age, age specific recommended amounts of sleep for infants, and allowing infants to have tummy time and non-confinement time beginning at 1 month of age. Additionally, Safe to Sleep® recommendations [11] were the topic of 3 of the 27 group meetings.

The PATE experimental curriculum of the intervention built upon the PAT curriculum by adding culturally tailored, maternal weight management and early childhood obesity prevention components. The PATE curriculum was guided by the theoretical underpinnings of the social cognitive theory which posits that learning occurs in a social context with a dynamic and reciprocal interaction of the person, environment, and behavior [12]. Intervention components aligned with the social cognitive theory included maternal modeling of positive health behavior. The PATE curriculum also was guided by the transtheoretical model of behavior change which integrates key constructs from other theories (i.e., decisional balance, self-efficacy, and process of change) into a comprehensive theory of change that can be applied to a variety of behaviors, populations, and settings [13]. Intervention components aligned with the transtheoretical model of behavior change included provision of information regarding the importance of eating healthfully (for both mother and infant) and being physically active (for both mother and infant) to positively affect attitudes and decisional balance. Additionally, to improve self-efficacy for engaging in these behaviors, discussions about overcoming personal barriers to and encouragement for healthful eating and being physically active were guided by the Parent Educators. Foundational elements from the Diabetes Prevention Program, including a flexible, culturally sensitive, individualized educational curriculum taught on a one-to-one basis, also were incorporated [14]. Further, anticipatory guidance and parenting support principals, elements of the Infant Feeding Activity and Nutrition Trial, were incorporated in the PATE curriculum [15]. Anticipatory guidance involves providing practical, developmentally appropriate, child health information to parents in anticipation of significant physical, emotional, and psychological milestones [16]. Parenting support emphasizes children’s psychological and behavioral goals, logical and natural consequences, mutual respect, and encouragement techniques [17].

Intervention components of the PATE arm included appropriate weight gain during pregnancy and weight management after pregnancy, nutrition and physical activity in the gestational (mother) and postnatal (mother and infant) periods, and parental modeling of healthful nutrition and physical activity behaviors. More specific to this paper, lesson topics included tummy and confinement time for infants (PM 1 visit), and family play time and decreased TV time (PM 6 visit). Lessons also included maternal weight gain (gestational) and loss (postnatal) charts for tracking participants’ body weight, infant growth charts, hands-on activities, instructional DVDs, and goal setting and barrier reduction for both diet and physical activity. Infants’ growth was plotted on World Health Organization (WHO) sex specific reference curves for length-for-age and weight-for-age percentiles.

Both arms of the intervention were delivered in the home to women beginning early in their second trimester of pregnancy by Parent Educators. Parent Educators were African American, college educated women residing in the target communities who had completed the onsite PAT foundational training program that included methods to build rapport and foster relationships with families and children. Parent Educators also were trained to deliver the nutrition and physical activity lessons and to collect data from participants by PhD level senior staff members. Home visits occurred monthly and were approximately 60–90 min in length for the PAT lessons, and approximately 90–120 min in length for the PATE lessons. The PATE lessons were longer in length due to the additional nutrition and/or physical activity information provided. Approximately 15 to 20 min of these visits were devoted to data collection. Both PAT and PATE participants received the same incentives at every visit. Gift cards were provided at the baseline and first and last postnatal visits. Other incentives included items such as diapers and baby bottles, books, and toys. A comprehensive description of the Delta Healthy Sprouts Project, including additional details regarding study methodology, lesson plan outlines, and Parent Educator training, has been published elsewhere [9].

### Measures

Participants’ knowledge and beliefs about infant activity and sleep were measured at baseline (gestational month 4 visit) and at study end (PM 12). The child activity knowledge and beliefs scale consisted of four true/false statements that can be found in Table 1. Items on these two scales were based on information provided to the participants during the course of the study. For each item, one point was given if the response was in the desired way (i.e., reflected the current state of knowledge about infant activity and sleep) and 0 points if the response was otherwise. The reliability of this scale was assessed in this study.

**Table 1:** Delta Healthy Sprouts child activity knowledge and beliefs questionnaire items and responses

Item	Question^c^	Baseline^a^		Study End^b^		Change		
		n	%	n	%	n_1_^d^	n_2_^e^	*P* ^f^
2a	It is OK to put a baby to sleep on his/her tummy. [F]	58	70.7	44	97.8	29	1	< 0.001
2b	It is OK to keep a baby less than 6 months old in a car seat or bouncy seat for most of the day. [F]	68	82.9	40	88.9	5	5	1.000
2c	Giving a baby tummy time (e.g. time on his/her tummy) should begin by 1 month of age. [T]	38	46.3	43	95.6	22	1	< 0.001
2d	A baby less than 12 months of age needs daily physical activity. [T]	67	81.7	41	91.1	8	2	0.058

^a^Gestational month 4 visit; number and percent of participants who chose correct response, *N* = 82

^b^Postnatal month 12 visit; number and percent of participants who chose correct response, *N* = 45

^c^Letter in brackets indicates correct response to statement; F = false and T = true

^d^Number of participants who chose incorrect response at baseline and correct response at study end (positive change)

^e^Number of participants who chose correct response at baseline and incorrect response at study end (negative change)

^f^*P*-values for McNemar’s test of symmetry for changes from baseline to study end. Bonferroni corrected significance level of α =0.013 used

Reports of their infants’ daily activities were collected from the participants (infants’ mothers) at each postnatal visit via electronic surveys. Measures included time spent unconfined and sleeping in a 24-h period, and whether or not the infant was put to sleep on his/her back (back to sleep) and had a regular bedtime. Non-confinement time (e.g., on play mat, in walker, in playpen) was used as a measure of infant physical activity. For this item, the five responses were less than 2 h, at least 2 h but less than 4 h, at least 4 h but less than 6 h, at least 6 h but less than 8 h, and 8 h or more. For infant sleep duration, the six responses were less than 8 h, at least 8 h but less than 10 h, at least 10 h but less than 12 h, at least 12 h but less than 14 h, at least 14 h but less than 16 h, and 16 h or more. For the back to sleep question, the three responses were yes always, yes sometimes, and no. For the regular bedtime question, the three responses were yes, no, and don’t know.

Anthropometric measures obtained on the participants at the baseline visit included height which was measured in duplicate using a portable stadiometer (model 217, seca, Birmingham, UK) and weight which was measured using a digital scale (model SR241, SR Instruments, Tonawanda, NY). Both measures were performed without shoes or heavy clothing. Pre-pregnancy body weight was self-reported. Body mass index (BMI) was calculated as weight (kg) divided by height (m) squared where height was averaged if the two measurements differed. Weight also was measured at each of the 17 subsequent (5 gestational and 12 postnatal) visits.

Anthropometric measures obtained on the infants included length and weight which were measured in duplicate if the two measures agreed or in triplicate if the two measures did not agree. For analytic purposes, the measures were averaged. Length was measured using an infantometer (model seca 416, seca, Birmingham, UK). Weight was measured using a digital scale (model SR241, SR Instruments, Tonawanda, NY) with the infant dressed in a diaper only and held in the mother’s arms (mother’s weight zeroed out). Infant length and weight were measured at each postnatal visit. Weight-for-length percentiles were calculated based on WHO reference growth curves for sex and age [18].

Participants provided self-reported information regarding socio-demographic characteristics (e.g., age relationship status, household size, education, employment, household income, insurance, prenatal care), Supplemental Nutrition Assistance Program (SNAP) and WIC participation, health history, and current health conditions at baseline. At the first postnatal visit, participants provided information regarding birth outcomes (e.g., delivery mode and infant gender, race/ethnicity, birth weight, and birth length). Details regarding other measures and questionnaire data that were collected but are not relevant to the present paper have been published elsewhere [9]. All measures and questionnaires were collected or administered by trained research staff (Parent Educators) using laptop computers loaded with relevant software (Snap Surveys, version 11.20, Snap Surveys Ltd., Portsmouth, NH).

### Statistical analysis

Statistical analyses were performed using SAS software. Results were considered significant at *p* ≤ 0.05 unless stated otherwise. Descriptive statistics, including means, standard deviations (SD), frequencies, and percentages, were used to summarize participants’ socio-demographic and anthropometric characteristics and their infants’ birth characteristics. Chi square tests of association or Fisher’s exact tests (categorical measures) and two sample t tests or Wilcoxon rank scores with exact *p*-values (continuous measures) were used to assess differences between postnatal study completers’ and non-completers’ baseline characteristics. Postnatal completers were defined as participants who had their PM 12 visit. Postnatal non-completers were defined as participants who had at least one visit in the postnatal period but did not complete the PM 12 visit.

To assess the reliability of the child activity knowledge and beliefs scale, the Kuder-Richardson 20 (KR-20) reliability measure was used since the item responses were dichotomous (true/false). KR-20 is analogous to Cronbach’s coefficient alpha with dichotomous data. A KR-20 value of 0.7 or above was considered acceptable reliability [19]. To assess changes in the individual items on this scale, McNemar’s tests of symmetry (suitable for paired nominal data) were used with Bonferroni correction factors applied (0.05/4 = 0.013). Associations between compliance with infant sleep recommendations and participants’ knowledge and beliefs about infant sleep were explored with Fisher’s exact test.

The National Sleep Foundation’s age specific recommendations, which are the result of consensus voting by an expert panel that included members of the American Academy of Pediatrics [20], were used to assess compliance with sleep duration recommendations. These recommendations were 14 to 17 h for newborns (0 to 3 months of age), which corresponded to survey responses 5 and 6 (14 to more than 16 h); 12 to 15 h for infants (4 to 11 months of age), which corresponded to survey response 4 and 5 (12 to less than 16 h); and 11 to 14 h for toddlers (1 to 2 years of age) which corresponded to survey responses 3 through 5 (10 to less than 16 h).

Generalized linear mixed models, using maximum likelihood estimation with the Laplace method, were used to test for significant effects of infant’s age (in months) and mothers’ knowledge and beliefs about infant activity on infant non-confinement time. These models also were used to test for significant effects of infant’s age (in months) and having a regular bedtime on compliance with sleep duration recommendations. Maximum likelihood estimation is an approach for handling missing data with longitudinal (repeated) measures. Infant non-confinement time was modeled using a multinomial distribution (suitable for ordinal measurements) with a cumulative logit function. Compliance with sleep duration recommendations was modeled using a binary distribution with a logit link function. Repeated measurement was modeled as a random effect using an unstructured covariance matrix and random intercepts. Because the item pertaining to keeping a baby in a car/bouncy seat most of the day was the only child activity knowledge and beliefs item that did not change in a positive direction and also because it had the lowest percentage of correct responses at study end, it was included in the infant non-confinement time model. Participants who responded correctly to this item at both baseline and study end were classified as “knowledge correct” and all others were classified as “knowledge incorrect.” Education level (less than or a high school education vs. at least some college or technical training) was included in the models as a covariate because it has been associated with infant behaviors [21].

Compliance with putting an infant to sleep on his/her back until 12 months of age and regular bed time were modeled as time-to-event data. The infant’s age (in months) at the time the participant first answered “no” or “yes sometimes” to the back to sleep question was used as the time to the event of interest (noncompliance with infant sleep position recommendation). Likewise, the infant’s age (in months) at the time the participant first answered “yes” to the regular bedtime question was used as the time to the event of interest. Observations were considered censored if the participant dropped from the study prior to her infant reaching 12 months of age or the infant reached 12 months of age without the respective events occurring.

Kaplan-Meier survival curves using the product-limit method were used to estimate median survival times. Survival analysis represents the most appropriate statistical methods available to handle time-to-event data, especially when censoring is involved [22]. Again maternal education level was included in both models as a covariate. The knowledge item corresponding to correct infant sleeping position (on his/her back) was not included as a covariate in the back to sleep model because of high correct response rates at both baseline and study end. Median survival times and 95% confidence limits were computed using a log-log transformation. Log rank tests were used to test for significant associations of survival time with the covariates.

## Results

The postnatal period retention rate was 85% (46/54). The mean number of postnatal visits was 10 (SD 3.2). Presented in Table 2 are maternal baseline socio-demographic and anthropometric characteristics of the postnatal cohort as well as characteristics of infants born to this cohort. The majority of participants were African American (96%), single (89%), receiving WIC (89%), and young (mean age 24 years, SD 4.8). Additionally, mean participant post-pregnancy BMI was in the obese category (31 kg/m^2^). The majority of participants’ infants were male (57%), fed formula within the first 24 h of birth (89%), and never breastfed (61%). Infant mean gestational age was 39 weeks and infant mean weight-for-height percentile at birth was 52%.

**Table 2:** Baseline socio-demographic, anthropometric, and birth characteristics of Delta Healthy Sprouts participants and their infants (*N* = 54)

Characteristic	n	%
Mothers		
Race		
African American	52	96.3
White	2	3.7
Relationship status		
Single^a^	48	88.9
Married	6	11.1
Education level		
≤ High school graduate	24	44.4
≥ Some college/technical	30	55.6
Employment status		
Full time/part-time	21	38.9
Unemployed (looking)	19	35.2
Homemaker/student	14	25.9
Smoker in household	16	29.6
Smoking status		
Current	2	3.7
Stopped before pregnancy	1	1.9
Stopped after became pregnant	1	1.9
Never	50	92.6
Medicaid health insurance	54	100.0
Receiving SNAP	41	75.9
Receiving WIC	48	88.9
Infants		
Male gender	31	57.4
Non-Hispanic ethnicity	53	98.1
Race		
African American	52	96.3
White	2	3.7
Fed formula within 24 h of birth	48	88.9
Ever breastfed	21	38.9
Premature (< 37 weeks gestation)^b^	7	13.0
	Mean	SD
Mothers		
Age (years)	23.6	4.84
Household size	3.9	1.58
Pre-pregnancy BMI^c^	28.9	7.91
Post-pregnancy BMI^d^	30.9	7.70
Infants		
Weeks gestation^b^	38.7	1.69
Birth weight (g)	3132.1	566.37
Birth length (cm)	48.6	2.92
Birth weight-for-length percentile^e^	51.9	37.45

*SNAP* Supplemental Nutrition Assistance Program, *WIC* Special Supplemental Program for Women, Infants and Children

^a^Includes 1 participant who indicated she is divorced

^b^Based on conception date (from online pregnancy calculator and using self-reported due date)

^c^Based on measured height and self-reported weight

^d^Based on weight measured at first postnatal visit

^e^Based on World Health Organization age- and sex-specific growth curves for children

Reliability was insufficient for the child activity knowledge and beliefs scale at baseline (KR-20 = − 0.29). Given the lack of reliability for this scale, questionnaire items were analyzed separately and not summed to create an overall measure of maternal knowledge and beliefs about infant activity and sleep. Summary and change statistics for these individual items are presented in Table 1. At baseline, participants’ knowledge and beliefs were generally in agreement with current recommendations with the exception of beginning tummy time at 1 month of age (less than half of the participants responded correctly to this item). Additionally, changes in responses for three of the items were in the direction hypothesized – proportionally more participants who answered the item incorrectly at baseline answered it correctly at study end as compared to participants who answered the item correctly at baseline and incorrectly at study end. Response changes were significant for two of the these three items – putting a baby to sleep on his/her tummy (false response) and tummy time should beginning by 1 month of age (true response). Proportions were approximately equal for the fourth item (keeping a baby in a car/bouncy seat most of the day), suggesting change occurred in both directions (positive and negative).

Median non-confinement time for infants increased steadily from at least 2 h but less than 4 h at PM 1 to 8 or more hours at PM 6. This median level of 8 or more hours of non-confinement time was maintained through PM 12 (data not shown in a table). Results of the generalized linear mixed models analysis for non-confinement time are presented in Table 3. Both infant age and participant knowledge concerning infant activity were significant explanatory variables in the model. For every one month increase in infant age, the odds of an infant having more non-confinement time increased by 10%. Additionally, infants with “knowledge correct” mothers had 17 times the odds of more non-confinement time as compared to infants with “knowledge incorrect” mothers. Because the confidence limit range for the knowledge variable is wide, this result should be interpreted cautiously.

**Table 3:** Generalized linear mixed model analysis for activity and sleep outcomes for Delta Healthy Sprouts participants’ infants

Outcome	Explanatory variable	OR	95% CL		*P*
Non-confinement time^a^	Infant age (in months)	1.1	1.06	1.20	< 0.001
	Knowledge correct^b^	17.4	3.31	91.02	0.001
	Knowledge incorrect^b^				
Sleep duration^c^	Infant age (in months)	1.3	1.23	1.40	< 0.001
	≤ High school education	2.2	1.11	4.38	0.024
	> High school education				

*OR* odds ratio, *CL* confidence limits

^a^Consisted of 5 ordinal responses; education was not a significant covariate

^b^Based on response to keeping an infant in a bouncy/car seat most of the day; item was coded as correct if the response was correct at both baseline and study end and incorrect otherwise

^c^Adherence to National Sleep Foundation age specific recommendations for amount of sleep; regular bedtime was not a significant covariate

Percentages of participants infants’ who met the National Sleep Foundation age specific sleep duration recommendations ranged from 10% at PM 2 to 87% at PM 12. For 9 out of the 12 study months, the majority (≥ 50%) of infants’ sleep duration met the recommended amounts (data not shown in a table). Results of the generalized linear mixed models analysis for meeting sleep duration recommendations also are presented in Table 3. Both infant age and education level were significant explanatory variables in the model. For every one month increase in infant age, the odds of compliance with sleep duration recommendations increased by 30%. Similarly, infants of participants with no more than a high school education had more than twice the odds of compliance with sleep duration recommendations as compared to infants of participants with at some postsecondary education.

Presented in Table 4 are results of the time-to-event analysis for compliance with infant sleep recommendations for the Delta Healthy Sprouts participants’ infants. Only 20% of the participants were compliant with the back to sleep recommendation for the first 12 months of their infant’s life. Conversely 94% of the participants’ infants were put to bed at a regular time at some point in the first 12 months of their life. Median time to noncompliance for the back to sleep recommendation was 7.8 months while median time to compliance for a regular bedtime was 4.2 months. Additionally, participants who did not consistently put their infant to sleep on his/her back for the first 12 months of life were as likely to correctly respond to the related child sleep statement (i.e., putting an infant to sleep on his/her tummy; false response) as participants who met the recommendation (97% vs. 100%, Fisher’s exact test *p* > 0.999; data not shown in a table).

**Table 4:** Time-to-event analysis for AAP infant sleep recommendations for Delta Healthy Sprouts participants’ infants

Recommendation	N	Not Met		Met		Dropped^a^		Survival time			P^b^
		n	%	n	%	n	%	Median	95% CL		
BTS until 12 months	54	38	70.4	11	20.4	5	9.3	7.8	5.6	8.5	0.820
Regular bedtime	54	0	0.0	51	94.4	3	5.6	4.2	3.6	5.1	0.387

*AAP* American Academy of Pediatrics, *BTS* back to sleep

^a^Participants dropped from the study prior to infant reaching age of recommendation or study end (censored event)

^b^*P*-value for log rank test for association of survival time with maternal education level

## Discussion

Delta Healthy Sprouts participants’ knowledge and beliefs concerning infant activity and sleep behaviors are presented in this paper. Results indicate that participants’ knowledge and beliefs about infant activity and sleep at baseline were generally in accordance with expert recommendations with the exception of tummy time. Most participants in this study were aware of expert recommendations concerning putting an infant to sleep on his/her back and providing an infant with daily non-confinement time or physical activity; less than half were aware that tummy time should begin by one month of age. However, at study end, all but one participant responded correctly to the item concerning tummy time. While it is possible that information provided to participants by their infant’s pediatrician or other healthcare professional was responsible for the participants’ increase in knowledge and beliefs, it is more likely that the increase can be attributed to the PAT Program. In an exploratory study by Koren and colleagues, only 15% and 26%, respectively, of the mothers surveyed immediately and two months postpartum reported receiving information about positioning their infant on his/her abdomen [23]. Further, providers surveyed in this study reported “random” counseling on tummy time when infants were approximately two to three months of age [23]. Taken together, these results suggest the need for increased awareness among infant caregivers concerning when to begin tummy time.

Similarly, educating parents and caregivers about unrestrained floor time for infants appears needed based on the lack of increased knowledge concerning keeping an infant in a bouncy/car seat most of the day (i.e., confined) in the current study. This is supported by the larger odds of more non-confinement time among infants with “knowledge correct” mothers as compared to infants with “knowledge incorrect” mothers. However, this result should be interpreted cautiously given the small number (*n* = 12) of participants who fell into the “knowledge incorrect” group. Given the success of the Safe to Sleep® public education campaign, a similar nationwide public education campaign to promote recommended awake positioning and adequate unrestrained floor time for infants may be needed to reach all populations within the US, with particular focus on caregivers of infants at highest risk for inactivity and childhood obesity.

Also presented in this paper is Delta Healthy Sprouts participants’ compliance with expert recommendations regarding infant sleep. While the majority of infants’ sleep duration was compliant with recommended amounts for three-fourths of the study period, the likelihood of compliance increased as the infants became older. Additionally, participants with no more than a high school education were over twice as likely to comply with infant sleep recommendations as compared to participants with some postsecondary education. This association has not been reported previously in the literature. Contrary to expectations, there was no association between regular bedtime and compliance with sleep duration for the infants in this study, although an association between regular bedtime routine and increased sleep duration had been reported previously [8]. The lack of association in the current study may be due to the majority of infants having a regular bedtime by approximately 4 months of age, compounded with the fact that all infants who remained in the study had a regular bedtime by approximately 12 months of age (i.e., lack of heterogeneity).

At the first postnatal visit, only two infants in this study were not consistently put to sleep on their back. Additionally, approximately half of the infants were consistently put to sleep on their back for the first eight months of their life. All 11 of the participants who put their infant to sleep on his/her back through 12 months of age and all but one of the 34 participants who did not follow this recommendation correctly answered the back to sleep knowledge item. Hence while the mothers in this study were aware of the back to sleep recommendation for their infants, over half of them chose not to follow the recommendation at some point during the first year of their infant’s life. Results from previous studies suggest that caregivers may not follow the sleep position recommendation due to reasons involving infant comfort and choking concerns [24]. Additionally, at approximately five to seven months of age, most infants can flip from their back to their stomach. Hence it is probable that mothers in the current study were not as concerned or consistent about proper sleep positioning once their infant was able to flip from back to front. Nonetheless, these results are concerning given racial disparities continue to exist for infant supine sleep (58% and 75% for African American and white populations, respectively) and Sudden Infant Death Syndrome (twice the rate in the African American population as compared to the white population) [24].

The longitudinal design of this study is one of its greatest strengths as is the novel use of time to event analysis for compliance with infant sleep recommendations. Additionally, the population studied is a strength because Southern and African American infants are at increased risk for rapid weight gain and non-supine sleep [3, 24]. Nonetheless data collection was not blinded and therefore a potential source of bias. However, it was not practically, logistically, or financially feasible to have a second set of blinded research staff whose purpose was solely to collect data. The study would have benefited from the use of a measure specific to tummy time and a more refined measure of infant sleep duration (e.g., intervals of one vs. two hours). Finally, arguably the most concerning limitation of this study is its small sample size which may have limited the ability to detect meaningful associations between maternal knowledge and beliefs about infant activity and sleep and infant activity and sleep behaviors.

## Conclusions

Baseline knowledge concerning infant activity and sleep recommendations was generally high in this cohort of rural, Southern, African American mothers. However, compliance with recommendations was not optimal suggesting the need for further intervention. Future research designed to improve infant activity and sleep outcomes should consider incorporating methods that not only increase caregivers’ knowledge and beliefs, but also positively impact caregivers’ actions.

## Abbreviations

BMI: Body mass index
KR-20: Kuder-Richardson 20
PAT: Parents as teachers
PATE: Parents as teachers enhanced
PM: Postnatal month
SD: Standard deviation
SNAP: Supplemental nutrition assistance program
US: United States
WHO: World Health Organization
WIC: Special supplemental program for women, infants, and children
